# Reconstruction of Glutathione Metabolism in the Neuronal Model of Rotenone-Induced Neurodegeneration Using Mass Isotopologue Analysis with Hydrophilic Interaction Liquid Chromatography-Zeno High-Resolution Multiple Reaction Monitoring

**DOI:** 10.1021/acs.analchem.2c04231

**Published:** 2023-02-03

**Authors:** Luojiao Huang, Nicolas Drouin, Jason Causon, Agnieszka Wegrzyn, Jose Castro-Perez, Ronan Fleming, Amy Harms, Thomas Hankemeier

**Affiliations:** † Metabolomics and Analytics Centre, Leiden Academic Centre for Drug Research, Leiden University, Leiden 2333 CC, Netherlands; ‡ SCIEX, Concord, Ontario L4K 4V8, Canada; § School of Medicine, National University of Ireland, University Rd, Galway H91 TK33, Ireland

## Abstract

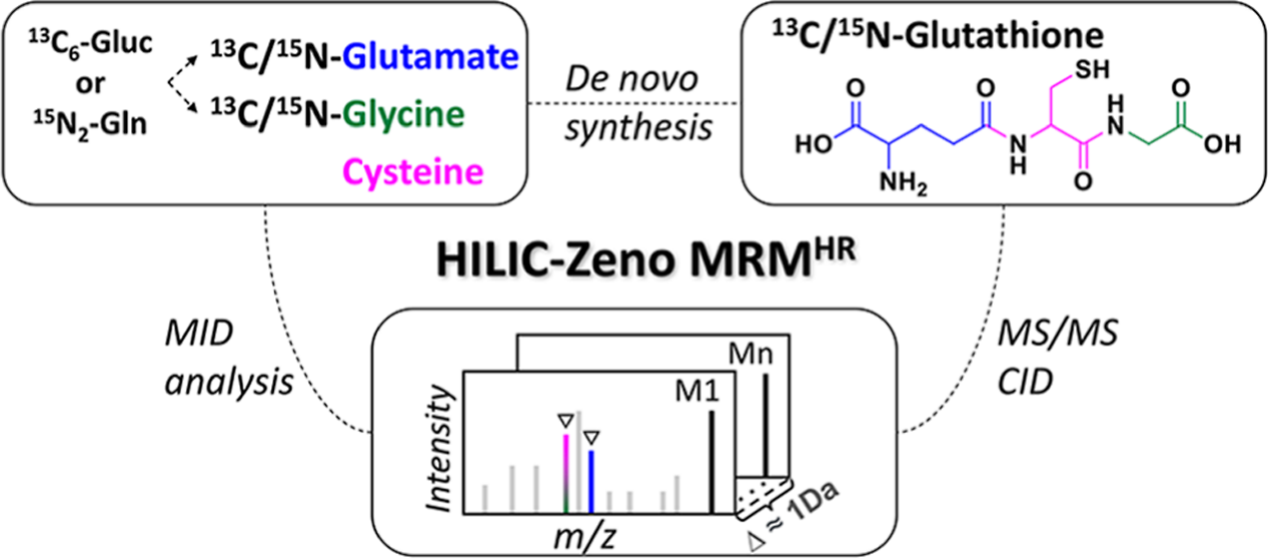

Accurate reconstruction of metabolic pathways is an important prerequisite for interpreting metabolomics changes and understanding the diverse biological processes in disease models. A tracer-based metabolomics strategy utilizes stable isotope-labeled precursors to resolve complex pathways by tracing the labeled atom(s) to downstream metabolites through enzymatic reactions. Isotope enrichment analysis is informative and achieved by counting total labeled atoms and acquiring the mass isotopologue distribution (MID) of the intact metabolite. However, quantitative analysis of labeled metabolite substructures/moieties (MS^2^ fragments) can offer more valuable insights into the reaction connections through measuring metabolite transformation. In order to acquire the isotopic labeling information at the intact metabolite and moiety level simultaneously, we developed a method that couples hydrophilic interaction liquid chromatography (HILIC) with Zeno trap-enabled high-resolution multiple reaction monitoring (MRM^HR^). The method enabled accurate and reproducible MID quantification for intact metabolites as well as their fragmented moieties, with notably high sensitivity in the MS^2^ fragmentation mode based on the measurement of ^13^C- or ^15^N-labeled cellular samples. The method was applied to human-induced pluripotent stem cell-derived neurons to trace the fate of ^13^C/^15^N atoms from D-^13^C_6_-glucose/L-^15^N_2_-glutamine added to the media. With the MID analysis of both intact metabolites and fragmented moieties, we validated the pathway reconstruction of de novo glutathione synthesis in mid-brain neurons. We discovered increased glutathione oxidization from both basal and newly synthesized glutathione pools under neuronal oxidative stress. Furthermore, the significantly decreased de novo glutathione synthesis was investigated and associated with altered activities of several key enzymes, as evidenced by suppressed glutamate supply via glucose metabolism and a diminished flux of glutathione synthetic reaction in the neuronal model of rotenone-induced neurodegeneration.

## Introduction

A metabolic network is a (sub)set of metabolic biochemical reactions known to take place in a living organism.^1^ Metabolomics studies quantitative changes in metabolite levels and can provide valuable insights into the understanding of disease origin, progression, and prognosis, as well as the effects and mechanism of pharmacological interventions.^2,3^ Metabolomics studies of Parkinson’s disease (PD) have suggested that energetic failure and increased oxidative stress are significant metabolic hallmarks in the neurodegeneration process.^4,5^ However, metabolic network robustness poses a challenge to the identification of pathway activities in response to perturbations^6,7^ because changes in metabolite consumption and production may not be accompanied by metabolite concentration changes. It is also difficult to distinguish between de novo synthesis and recycling of the existing metabolite pool, which reflects the activity of different metabolic pathways.

Isotope tracing techniques allow one to trace the incorporation of heavy atoms (stable or radioactive^8^) into downstream intermediates from a given labeled precursor. It is an excellent way to monitor pathway activity and has been successfully applied to different levels of organism studies, such as *ex vivo* tissues,^9,10^ in vivo animal models,^9−11^ and in vitro cellular culture.^12^ Mass spectrometry (MS) has become the principal technique used for the analysis of stable isotope-labeled metabolites. It requires only a small amount of sample, manifests excellent detection sensitivity, and can provide structural information on multiple compounds simultaneously. Labeled distributions of intact molecules can be obtained via MS measurements, which consist of a set of mass isotopologue abundances. Mass isotopologue distribution (MID) analysis at the MS^1^ level has been generally used in tracer-based metabolomics studies for tracing labeled enrichment through intermediates and probing pathway activity.^13−16^ Subsequently, more attention has been paid to acquiring substructural information on labeled isotopologues and improving metabolic flux interpretation.^17,18^

Tandem MS-based approaches, using multiple reaction monitoring (MRM), can reveal the isotope labeling states of selected precursor and product ions by including all possible combinations in the transition pairs.^9,19−21^ This method is popular for achieving good performance in metabolite quantification. However, it still shows technical drawbacks in measuring stable isotope-labeled metabolites in a broader metabolomic scope. The number of transition pairs considerably increases with an increasing metabolite atom number, leading to a longer cycle time and fewer data points per peak, as well as less accurate quantification and lower sensitivity for low-abundance isotopologues. In the case for phosphorylated metabolites, compared to PO_3_^–^ or H_2_PO_4_^–^ ions, carbon-containing product ions carry more structural information and are more useful for atom tracing over intersecting pathways. However, they are generally in very low abundance, which requires a longer dwell time for each transition pair to reach good sensitivity.^22^

To overcome these difficulties, advanced tandem MS-based approaches have been developed recently. The MRM methods on triple quadrupole instruments with dynamic modification of the mass filter resolution for precursor or product ions can effectively minimize total MRM transitions, enabling the detection of intact and fragmented metabolite isotopologues with good quantification accuracy in two separate runs.^22^ Based on a quadrupole linear ion trap instrument, a new liquid chromatography (LC)–mass spectrometry (MS)/MS acquisition method, and a novel isotope recapitulation algorithm (MID Max), the coverage of intact and fragmented metabolite isotopologues has been further extended by combining MRM and an enhanced data-dependent product ion scan type in a single run.^23^ Parallel reaction monitoring (PRM) based on high-resolution MS was able to obtain intact and fragmented isotopologue distributions in high resolution within a single analytical run, resulting in a significantly lower cycle time compared to MID Max.^24^ Other tandem MS-based approaches in high resolution via data-independent acquisition techniques are also available, such as SWATH fragmentation over stacked mass isolation windows on a QqTOF MS^25−27^ and all-ion fragmentation within a wide, predefined mass window on an Orbitrap Fusion Tribrid MS.^28^ When using the SWATH technique, erroneous MID quantification was found for precursors positioned on the margins of two neighboring windows.^26^ This requires special attention to properly design Q1 isolation windows for target metabolite quantification. Jaiswal et al. suggested employing two different SWATH programs to achieve good MID quantifications corresponding to 19 intermediate metabolites by ensuring all precursor isotopologues fall into a single window in one of the programs.^26,29^ Compared to PRM, the co-fragmentation of all isotopologues of certain metabolites in a single mass window showed higher sensitivity in quantifying precursor and fragment isotopologues of low abundance.^29^ However, there is no direct spectral connection between a precursor and its corresponding fragments, making it difficult to determine the detailed positioning of labeled atoms within a particular precursor isotopologue. This type of tandem isotopologue distribution, to be noted, has shown strong benefits for improving metabolic flux analysis.^30,31^

Metabolic pathway reconstruction of central carbon metabolism and its connected de novo synthesis pathways is critical for understanding the consecutive reaction changes from energy failure toward oxidative stress in Parkinson’s disease. Therefore, to facilitate reconstructing metabolite transformations along these pathways and offer a comprehensive picture of metabolic regulation using both intact and fragmented metabolite isotopologues, we need high-sensitivity detection but also high data quality for structural elucidation of the MS^2^ spectra. In this work, we present hydrophilic interaction liquid chromatography (HILIC)–multiple reaction monitoring (MRM^HR^) using Zeno trap pulsing, a recently introduced system of trapping fragment ions prior to the time-of-flight (TOF) injection. This method combines the advantages of HILIC for wide coverage of polar metabolome analysis and the Zeno trap-enabled technique for duty cycle improvement.^32^ We compared the performance of the Zeno method to that of the SWATH method and MRM^HR^ (general PRM) with regards to the aspects of sensitivity, accuracy, and fragmentation reproducibility in MID analysis. We further applied the HILIC-Zeno MRM^HR^ method to a classic neuronal model of rotenone-induced neurodegeneration and revealed diverse flux regulations via glucose and glutamine metabolism into glutathione metabolism related to neurodegeneration.

## Experimental Section

### Chemicals and Reagents

Standards were purchased from Sigma-Aldrich (Zwijndrecht, The Netherlands) and Fluka (Seelze, Germany). The tracer substances D-^13^C_6_-glucose (99% isotopic purity) and L-^15^N_2_-glutamine (98% isotopic purity) were purchased from Cambridge Isotope Laboratories (Tewksbury, MA, USA). Acetonitrile in LC–MS grade and chloroform in HPLC grade were purchased from Biosolve B.V. (Valkenswaard, The Netherlands). Methanol in Ultra-LC-MS grade was purchased from ActuAll (Oss, The Netherlands). Milli-Q Ultra-pure water was obtained from a Merck Milli-pore A10 purification system (Raleigh, USA). Ammonium formate (≥99.995% trace metal basis) and rotenone were purchased from Sigma-Aldrich (Zwijndrecht, The Netherlands). Ammonium hydroxide (28–30 wt % solution of ammonia in water) was purchased from Acros Organics (Geel, Belgium).

### Standard Solutions and Cell Culture Medium

Individual stock solutions of 40 standards were made with 50% MeOH or pure water in 1 mg/mL and stored in −80 °C (Table S1). Mixed standard solutions were prepared at the concentrations of 20, 15, 10, 7.5, 5.0, 2.5, 1.25, 0.5, and 0.1 μg/mL with 50% MeOH as the dilution solution. According to an adapted protocol from Reinhardt,^33,34^ a basal neuron culture medium, N2B27, was made by mixing equal amounts of neurobasal medium (Invitrogen/Life Technologies) and Dulbecco’s modified Eagle’s medium/F12 medium (Invitrogen/Life Technologies) and adding 1% penicillin/streptomycin (Life Technologies), 2 mM l-glutamine (Life Technologies), 1:100 B27 supplement without vitamin A (Life Technologies), and 1:200 N2 supplement (Life Technologies). Maintenance medium was made of high-glucose N2B27 medium supplemented with 150 μM ascorbic acid (Sigma-Aldrich), 0.5 μM PMA (Enzo Life Sciences), and 3 μM CHIR (Axon Medchem). Differentiation medium was made of high-glucose N2B27 medium supplemented with 200 μM ascorbic acid, 0.01 ng/μL BDNF (PeproTech), 0.01 ng/μL GDNF (PeproTech), 0.001 ng/μL TGFβ-3 (PeproTech), 2.5 μM dbcAMP (Sigma-Aldrich), and 1 μM PMA (absent after 6 days of differentiation).

^13^C-labeled maintenance medium and differentiation medium were made by replacing 20.4 mM glucose with the same amount of D-^13^C_6_-glucose so that the pool size of glucose remains the same. ^15^N-labeled maintenance medium and differentiation medium were made by replacing 2 mM glutamine with the same amount of L-^15^N_2_-glutamine so that the pool size of glutamine remains the same.

### Cell Culture

For method development and evaluation, the iPSC-derived human neuroepithelial stem cells (hNESCs) were cultured on a 12-well plate at a density of 300,000 cells/well. Five wells of hNESCs were incubated with maintenance medium containing D-^13^C_6_-glucose, and another five wells of hNESCs were incubated with maintenance medium containing L-^15^N_2_-glutamine. Two wells of hNESCs were incubated with normal maintenance medium. The incubation time was 24 h. The ^13^C- and ^15^N-labeled cellular samples were used as labeled reference samples for method evaluation.

Next, for method application, hNESCs were cultured and differentiated into mid-brain neurons on a 12-well plate at a density of 180,000 cells/well by following the established protocol.^33,34^ After 21 days of neuron differentiation and maturation, we switched the normal differentiation medium into ^13^C- or ^15^N-labeled differentiation medium. In the ^13^C-labeling culture with D-^13^C_6_-glucose (^13^C_6_-Gluc), five replicates of labeled neuron culture were designed for the healthy group and the rotenone (200 nM) exposure group, respectively, and were accompanied by one unlabeled neuron culture within each group. The same sample design was applied in the ^15^N-labeling culture with L-^15^N_2_-glutamine (^15^N_2_-Gln). Differentiated neurons were under incubation with tracers for 24 h and reached isotopic labeling stationarity in metabolites. For cell quenching, ice cold 200 μL of 80% MeOH was added immediately after removing the spent medium and washing with phosphate buffered saline (Gibco/Life Technologies). The quenched cell samples were harvested into a new Eppendorf tube. Cellular samples were fast frozen into liquid nitrogen and stored in the −80 °C freezer until LC–MS measurement. Results from unlabeled neurons were used for qualitative peak confirmation during data analysis.

### Sample Preparation

Cell samples were lysed with sonication after one freeze–thaw cycle, vortexed, and then centrifuged at 16000*g* at 4 °C for 10 min. Cell pellets were collected to measure the protein content using a bicinchoninic acid assay (Thermo Fisher Scientific Inc, United States). Supernatants were transferred into clean 1.5 mL Eppendorf tubes and evaporated to dryness in a Labconco SpeedVac (MO, United States). Each sample was reconstituted with 60 μL of ice-cold methanol/water (80%/20%; v/v). 50 μL of the reconstitution volume was collected and transferred into a new Eppendorf tube. The leftover volume was pooled together as a quality control (QC) sample for each group. Next, the reconstituted samples and QC samples were processed with liquid–liquid extraction by adding 40 μL of ice-cold methanol/water (80%/20%; v/v), 45 μL of ice-cold Milli-Q water, and 65 μL of ice-cold chloroform, then followed with mixing and vortexing for 5 min and centrifuging at 16000*g* 4 °C for 10 min. 130 μL of the aqueous phase was transferred into a new Eppendorf tube and extracted again by adding 25 μL of ice-cold methanol/water (50%/50%; v/v) and 65 μL of ice-cold chloroform, then followed with mixing and vortexing for 5 min and centrifuging at 16000 rcf, 4 °C for 10 min. Finally, 140 μL of the aqueous phase was transferred and taken to dryness. The residue was reconstituted with 50 μL of ice-cold methanol/water (50%/50%; v/v) as the final injection solution for LC–MS measurement. A series of diluted reference samples was prepared by diluting the ^13^C-labeled reference sample twofold (DF_2x) and threefold (DF_3x) with the injection solvent of methanol/water (50%/50%; v/v).

### LC–MS Measurement

Chromatographic separation was performed using the SeQuant ZIC-c HILIC HPLC column (2.1 mm × 100 mm, 3.0 μm, Merck, Darmstadt, Germany) on a Shimadzu Nexera Ultra high-performance liquid chromatograph (LC) (Duisburg, Germany). The LC method was adapted from a previously described method.^35^ Mobile phase A consists of 90% acetonitrile and 10% water with 5 mM ammonium formate, and mobile phase B consists of 10% acetonitrile and 90% water with 5 mM ammonium formate. The injection volume was 3 μL. The flow rate was 0.5 mL/min, and the gradient was as follows: 0 min–0% B, 2 min–15% B, 5 min–21% B, 7.5 min–26% B, from 10 to 11 min–40% B, from 11.5 to 18 min–0% B. The MS analyses were performed on a SCIEX ZenoTOF 7600 system (Darmstadt, Germany) with a TwinSpray Turbo V ion source and operated in negative electrospray ionization. The following ion source parameters were applied: a spray voltage of 4.5 kV, a capillary temperature of 400 °C, ion source gas of 1 20 psi, ion source gas of 1 50 psi, curtain gas of 25 psi, and CAD gas of 7 psi.

A SWATH acquisition method was able to fragment all isotopologues within stacked mass windows over the chromatographic run. Each MS cycle starts with a survey TOF MS scan in 100 ms from 50 to 700 Da using the declustering potential (DP) at −80 eV and collision energy (CE) at −5 eV, followed by a fixed Q1 isolation window setting. The Q1 isolation strategy covered a mass range of *m*/*z* 60–690 with a 40 Da window width for Q1 isolation (overlap 1 Da). It allowed all possible isotopologues of each target metabolite to be fragmented in the same window. The SWATH scan accumulation time was 85 ms, and each cycle time was 1.576 s using DP at −80 eV and CE at −30 eV ± 20 eV. We also tested additional SWATH window settings where the targeted isotopologues fell in two adjacent windows. The curated window settings can be seen in the Supporting Information, Table S2 and Figure S1.

The MRM^HR^ acquisition method consisted of the same TOF MS scan applied in the SWATH acquisition method, followed by MS/MS scans of the inclusion precursors with unit Q1 isolation and scheduled retention times. The targeted precursors are different for ^13^C- and ^15^N-labeled sample analysis. Based on the measurement of ^13^C and ^15^N-labeled reference cell samples, in total, 180 precursor ions from 25 metabolites were targeted in the ^13^C-labeling MRM^HR^ acquisition method, and 55 precursor ions from 15 metabolites were targeted in the ^15^N-labeling MRM^HR^ acquisition method (Tables S3–S4). DP at −80 eV and CE at −30 eV ± 20 eV were applied to all precursor ions to have a fair comparison to SWATH acquisition. The Zeno MRM^HR^ acquisition method was designed based on the MRM^HR^ acquisition method and set with the Zeno-trap on-demand above the collision-induced dissociation intensity threshold of 2000 cps.

### Data Analysis

Qualitative data analysis was performed using the SCIEX OS Explorer processing tool. The fragmentation behavior analysis used the online databases Metlin^36^ and mzCloud (https://www.mzcloud.org/) as references and was confirmed with our in-house MS^2^ database using analytical standards (see Supporting Information). Quantitative data analysis was performed using the SCIEX OS Analytics processing tool. The peak areas of metabolite isotopologues in the MS^1^ and MS^2^ levels were integrated and further corrected for the natural stable isotope abundance using software IsoCor.^37^ MID represent the relative abundance of different mass isotopologues and are reported as isotopologue fractions. The ^13^C/^15^N enrichment refers to the mean content of isotopic tracer in the metabolite. It was calculated by the formula ME = Σ_*i*=1_^*n*^Mi × *i*/*n*, where Mi is the proportion of isotopologues with *i*^13^C atoms for a metabolite containing *n* carbon atoms. Tandem MID analysis was calculated based on the primary MID and further applied with the secondary distribution ratio of isotopomers.

## Results and Discussion

To meet the study goal of capturing both intact and fragment-labeled isotopologue distributions of metabolites, we developed a MS/MS quantification method based on Zeno MRM^HR^ acquisition coupled to a HILIC separation method. Given the fact that a high number of transitions results in fewer scan points for each transition in the same retention time window, we first optimized the mobile phase gradient of a previously developed HPLC method utilizing a ZIC-c HILIC column for polar metabolite analysis.

### HILIC-Zeno MRM^HR^ Method Development

In total, 40 polar metabolites derived from primary carbon metabolism, glutathione metabolism, and purine and pyrimidine metabolism achieved good chromatographic separation for standard solutions (Table S1, Figure S2). For the HILIC-Zeno MRM^HR^ method, 25 selected metabolites including all possible isotopic states were included in the ^13^C-labeling MS^2^ fragmentation method, and 15 metabolites including all possible isotopic states were included in the ^15^N-labeling MS^2^ fragmentation method. The selected metabolites were critical intermediates in their relevant metabolic pathways and were detected in labeled states with a TOF MS scan in the reference ^13^C (^15^N) cellular sample set. Finally, for metabolites eluting at retention times between 4 and 6 min, where the peak density is the highest, the method ensured a minimum of eight scan points across chromatographic peaks at the base (Figure S3). Under both MS^1^ and MS^2^ levels, the method exhibited good linearity for targeted metabolites, with correlation coefficients mostly above 0.99 (Table S5).

With proper isolation window settings, SWATH methods have been reported for MID quantification of targeted metabolites and their fragments with good sensitivity and small error.^26^ To confirm the impact of entire or partial isotopologue coverage in one Q1 window, as well as the impact of overlapping windows offering partial isotopologue coverage, several SWATH acquisition methods with various mass window settings were evaluated. Our results showed that the quantification of isotopologues that span two windows suffers from peak intensity loss and reduced fidelity (Figure S1). As a reference method for our subsequent method comparisons, we selected a SWATH method with a fixed Q1 isolation window to encompass the intact MID of target metabolites.

### Evaluation of Sensitivity and Isotope Fidelity

**Figure 1:**
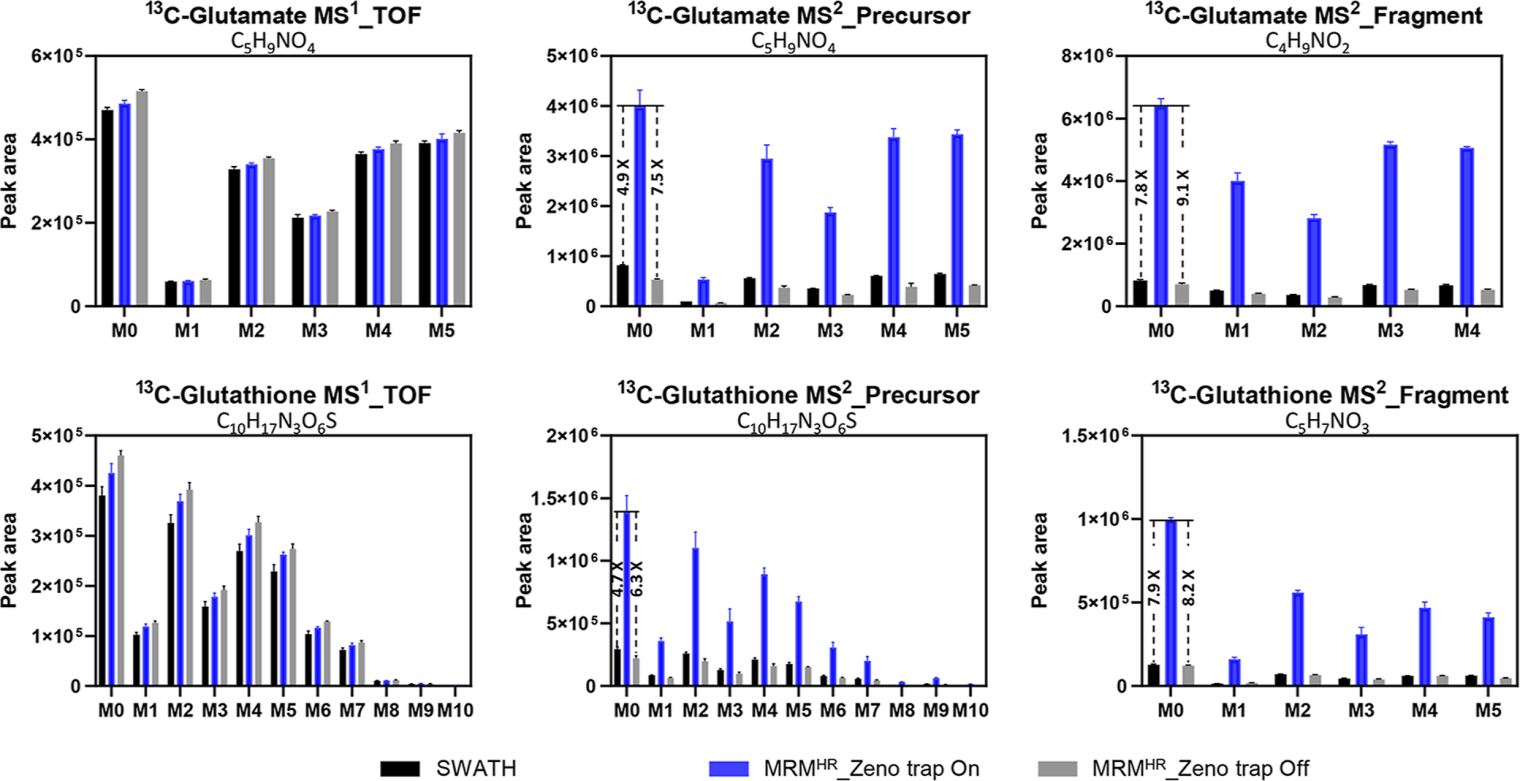
Sensitivity comparison at the MS^1^ TOF level and MS^2^ fragmentation level among SWATH, MRM^HR^, and Zeno MRM^HR^ acquisition for ^13^C-labeled isotopologue analysis (*n* = 3). At the MS^2^ level, each precursor isotopologue was quantified using the peak area of the residual precursor ion extracted from its MS/MS scan window. Each fragment isotopologue was quantified by summing the peak areas of the same fragment ion extracted from multiple MS/MS scan windows.

Next, we evaluated the HILIC-Zeno MRM^HR^ method on the quantification performance for precursor and fragment isotopologues and compared these to the HILIC–MRM^HR^ and HILIC–SWATH methods. As shown in Figure 1, in the MS^1^ TOF level, minor differences in the peak area were detected because of the slight differences in the MS scan cycle duration between SWATH, Zeno MRM^HR^, and MRM^HR^ methods. Whereas, in the MS^2^ level, a significant signal improvement with the Zeno MRM^HR^ method was observed for all fragment and residual precursor isotopologues in comparison to SWATH and MRM^HR^ methods. The Zeno trap enables almost 100% duty cycles in MS/MS, resulting in signal gains without loss of mass accuracy or resolution.^38^ The Zeno trap method improved the signal for fragment ions more than for their precursor ions, mostly because of a higher Zeno pulsing gain for lower masses. In comparison to SWATH, the ^13^C-glutamate precursor increased 4.9-fold, while its fragment increased 7.8-fold; the ^13^C-glutathione precursor increased 4.7-fold, and its fragment increased 7.9-fold with the Zeno trap enabled. Significant sensitivity increases were also seen using the test results for ^15^N-labeled reference cell samples (Figure S4). Precursor ions of ^15^N-glutamate showed an increase of 6.6-fold, and fragment ions of ^15^N-glutamate showed an increase of 8.4-fold compared to the SWATH method.

**Figure 2:**
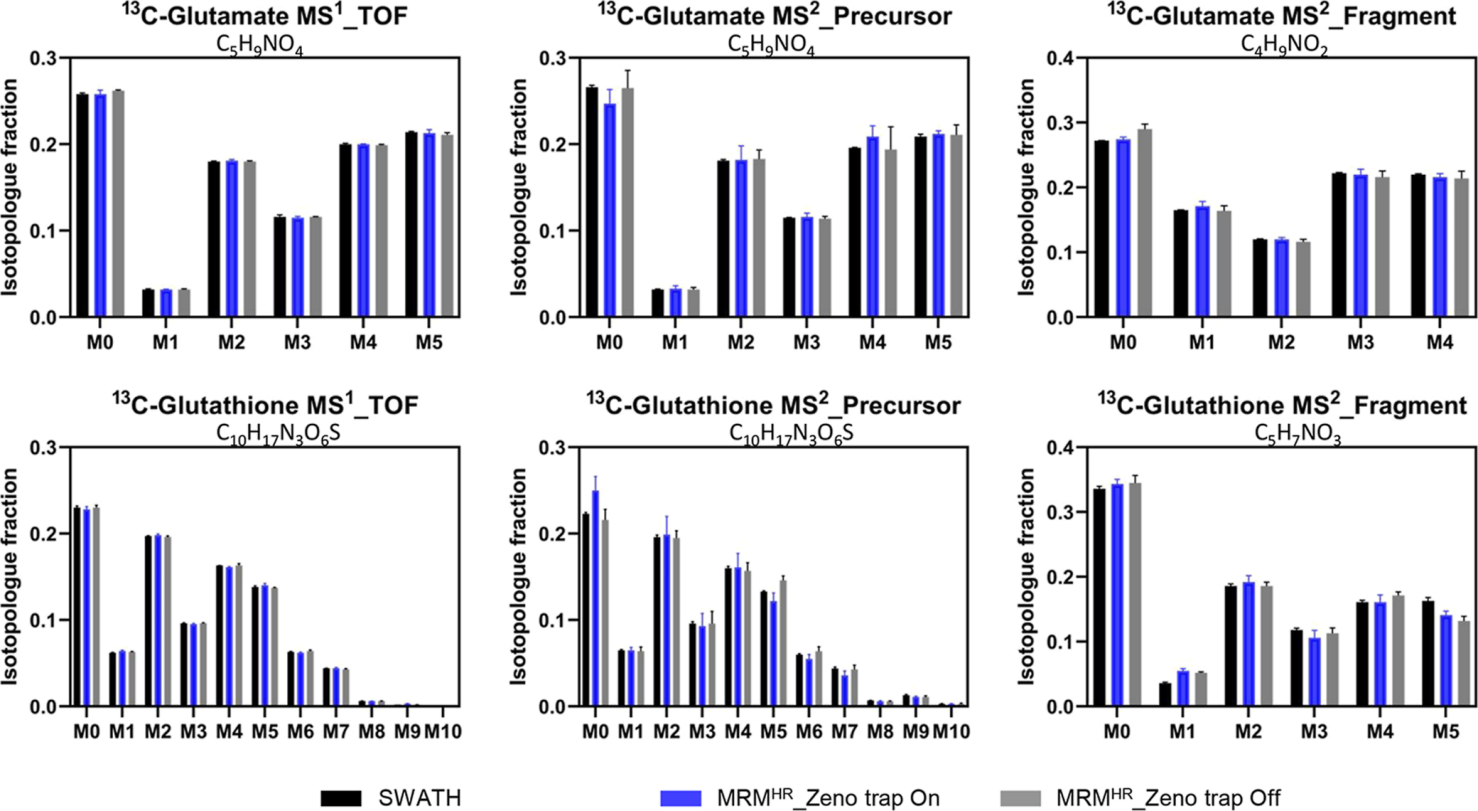
Accuracy comparison at the MS^1^ TOF level and the MS^2^ fragmentation level among SWATH, MRM^HR^, and Zeno MRM^HR^ acquisition for ^13^C-labeled isotopologue distribution analysis (*n* = 3).

Moreover, the sensitivity gain still maintains an accurate MID. For metabolites containing 5 carbons (glutamate), or 10 carbons (glutathione), shown in Figure 2, the SWATH, Zeno MRM^HR^, and MRM^HR^ methods shared the same TOF MID results; in addition, the precursor MID was in line with the TOF MID. This provided confidence for further investigation of fragment MID. At the MS^2^ fragment level, the Zeno MRM^HR^ method preserved identical ^13^C isotopologue distributions as the other methods. No artifacts were introduced during Zeno trap pulsing in the Zeno trap. Likewise, for metabolites with one nitrogen (glutamate) or three nitrogens (glutathione) at both MS^1^ and MS^2^ levels, the Zeno MRM^HR^ results maintained identical ^15^N isotopologue distribution as the other two methods (Figure S5). Overall, the HILIC-Zeno MRM^HR^ demonstrated its strong advantages in labeled mass isotopologue analysis in terms of detection sensitivity and isotope fidelity at the MS^2^ level.

### Evaluation of MID Quantification Reproducibility

**Figure 3:**
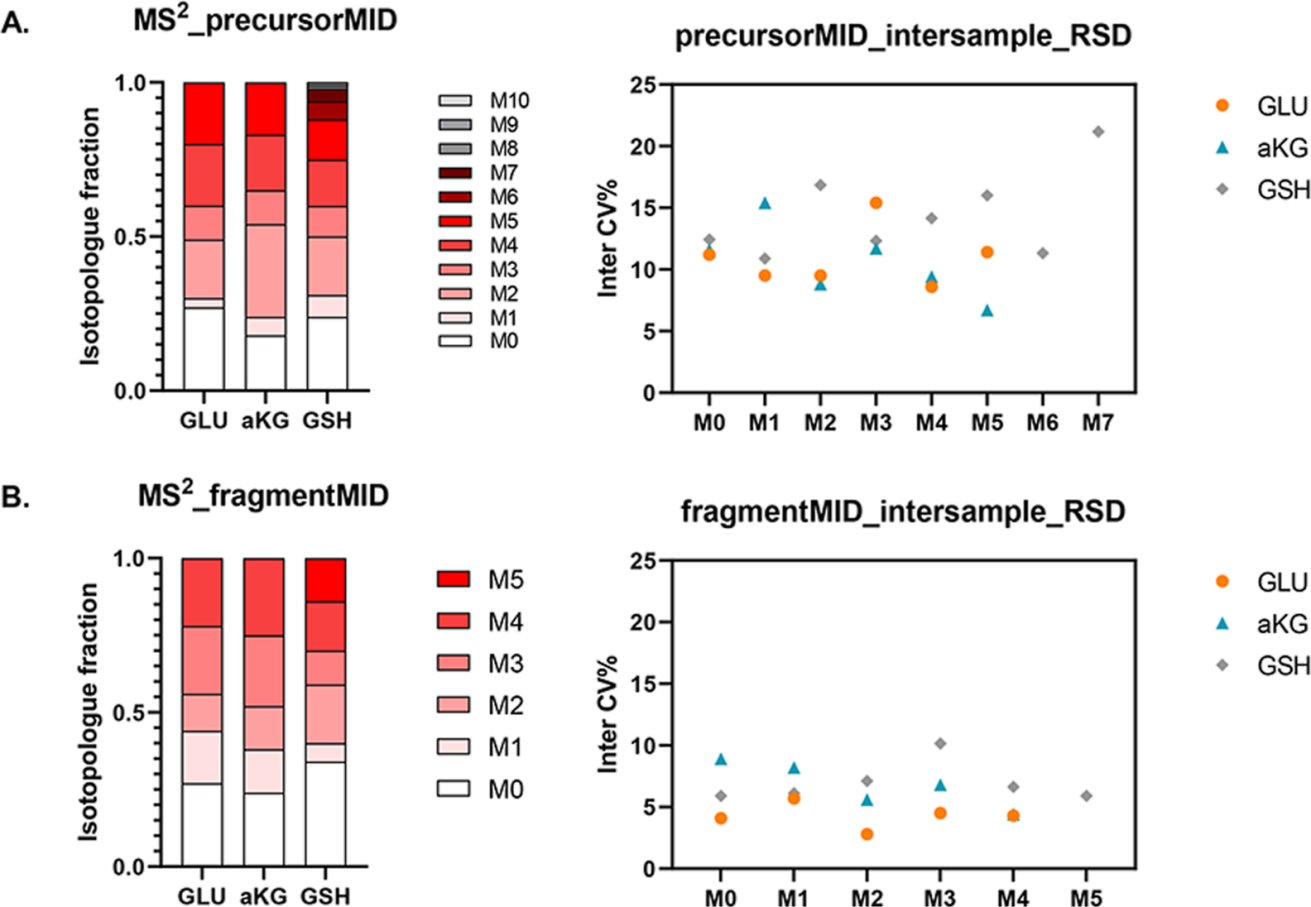
Inter-sample reproducibility of ^13^C MIDs based on one reference sample set, including no dilution, twofold dilution, and threefold dilution. Each sample was injected three times. (A) Isotopologue fractions for precursor ions on average (left, *n* = 9) and their corresponding RSD (right, *n* = 9). (B) Isotopologue fractions for fragment ions on average (left, *n* = 9) and their corresponding RSD (right, *n* = 9). Glutamate: GLU; ketoglutarate: aKG; glutathione: GSH.

In a typical cell culture, the harvested quantity of cells often varies between replicated culture wells. Nonetheless, regardless of variations in total cellular content, isotopologues in fractions should be constant among replicates of a group assuming a consistent metabolic state. We further evaluated the HILIC-Zeno MRM^HR^ method reproducibility in MID quantification for inter-sample analysis. A set of ^13^C reference samples in undiluted form (DF_1x), twofold dilution (DF_2x), and threefold dilution (DF_3x) was evaluated to imitate the effect of varied metabolite concentrations across samples. The average protein content corresponding to DF_1x, DF_2x, and DF_3x samples was 38.0, 19.0, and 12.7 μg, respectively. As shown in Figure 3, for the metabolites glutamate, ketoglutarate, and glutathione, precursor MIDs had relative standard deviations (RSDs) between 6.7 and 21.2%, and fragment MIDs had RSDs between 2.8 and 10.1% across DF_1x, DF_2x and DF_3x samples. Fragment MID exhibited better quantification reproducibility than precursor MID. The corresponding MID data in detail can be found in Table S6. Overall, the MID quantification of the HILIC-Zeno MRM^HR^ method over inter-sample analysis demonstrated a reproducibility RSD of less than 25%. The performance of MS/MS fragmentation with the Zeno trap enabled showed good robustness to varied sample concentrations.

### Tandem Mass Isotopologue Distribution Analysis of Glutamate

**Figure 4:**
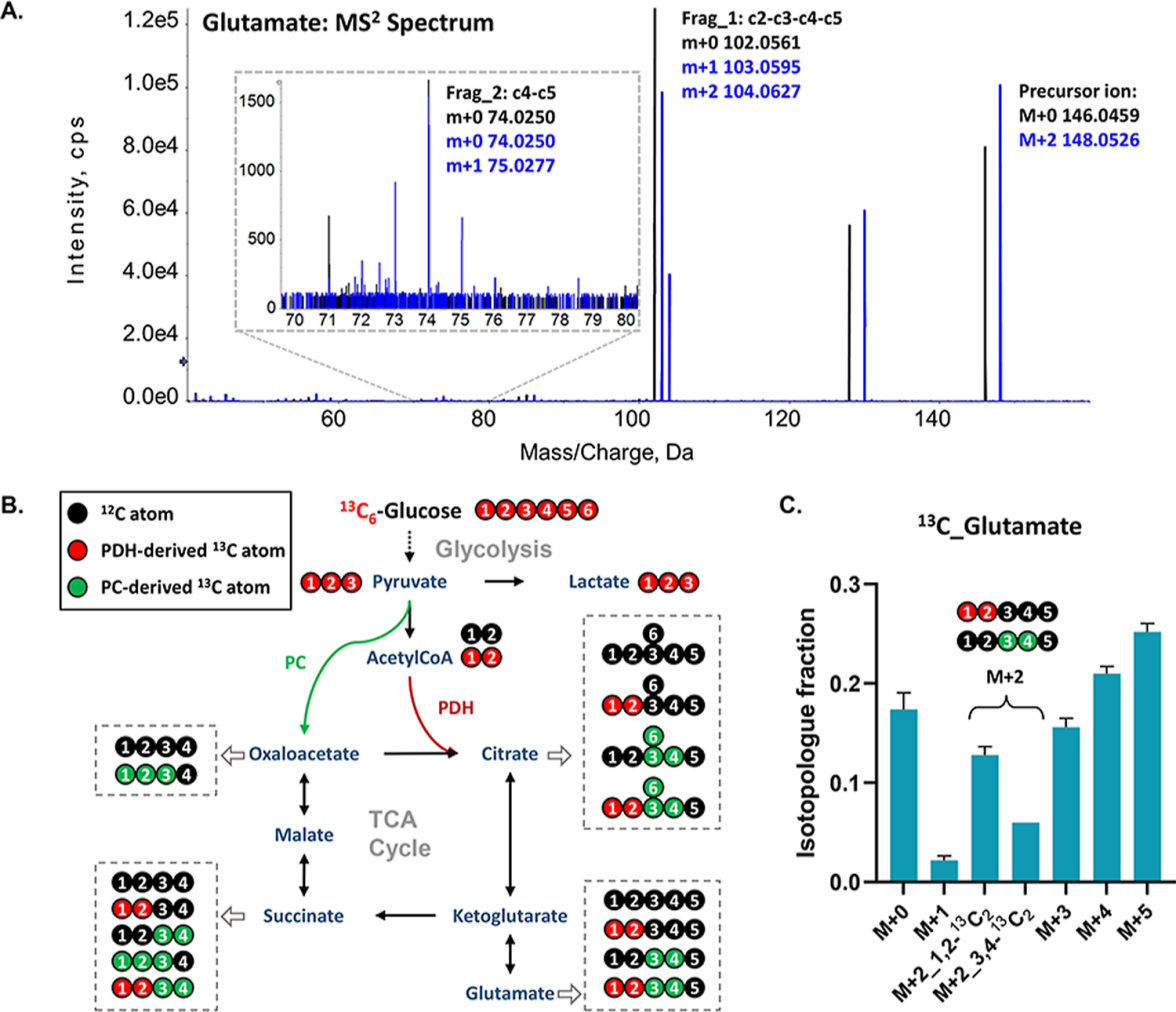
Structural elucidation of ^13^C_2_-glutamate through analyzing the labeling pattern of annotated moiety fragment_1 (Frag_1: c2-c3-c4-c5) and fragment_2 (Frag_2: c4-c5) at the MS^2^ level. (A) MS^2^ spectrum of the monoisotopic and ^13^C_2_ isotopologue peak of ^13^C-glutamate. (B) ^13^C atom tracking derived from D-^13^C_6_-glucose into glycolysis and the TCA cycle. The expected ^13^C-labeling patterns for the intermediates, acetylCoA, citrate, glutamate, succinate, and oxaloacetate, via PDH and PC pathways were deduced and depicted in red and green, respectively. The first turn of PDH-initiated and PC-initiated ^13^C-labeling results were displayed. (C) Tandem MID of ^13^C-glutamate measured in healthy neurons.

One unique advantage of the HILIC-Zeno MRM^HR^ method is its capacity to resolve the labeling positional information for a particular isotopologue. To exemplify this, we used this method to distinguish two sets of ^13^C-labeling positions in the ^13^C_2_-glutamate isotopologue derived from D-^13^C_6_-glucose. Figure 4A shows the detected M + 0 precursor ion of glutamate and its produced fragments labeled in black, and the M + 2 precursor ion and its produced fragments labeled in blue. Fragment_2 produced from M + 2 isotopologue showed no labeled m + 2 peak, indicating that simultaneous labeling of two ^13^C atoms at the C4 and C5 positions was impossible. As illustrated in Figure 4B, glutamate derived from ^13^C_6_-glucose after one round of ^13^C incorporation via the tricarboxylic acid (TCA) cycle can result in two ^13^C atoms at the C1 and C2 positions via pyruvate anaplerosis (PDH) and two ^13^C atoms at the C3 and C4 positions via the pyruvate carboxylase (PC) pathway.^39,40^ By analyzing the corrected peak area ratio between the m+1 and m+2 peaks of fragment_1, we could further determine the distribution ratio between 1,2-^13^C_2_-glutamate and 3,4-^13^C_2_-glutamate, and generate a tandem MID of ^13^C-glutamate in Figure 4C (Table S7). Healthy mid-brain neurons exhibited a relatively higher flux via PDH activity than PC activity.

### Reconstruction of Glutathione Metabolism in Mid-Brain Neurons

Glutathione (GSH), one of the intracellular antioxidants, can protect cells by neutralizing reactive oxygen species and converting itself into its oxidized form (GSSG).^41^ In modulating redox homeostasis, de novo GSH synthesis was reported to play a more critical role than recycling GSSG.^42,43^ Rotenone is known as a classic toxin for causing dopaminergic degeneration by inducing oxidative stress. To better distinguish the metabolic change of glutathione metabolism via de novo synthesis among intersecting pathways, the HILIC-Zeno MRM^HR^ method was applied to measure the polar ^13^C/^15^N-metabolome from healthy and rotenone-treated mid-brain neurons with D-^13^C_6_-glucose/L-^15^N_2_-glutamine as a tracer.

**Figure 5:**
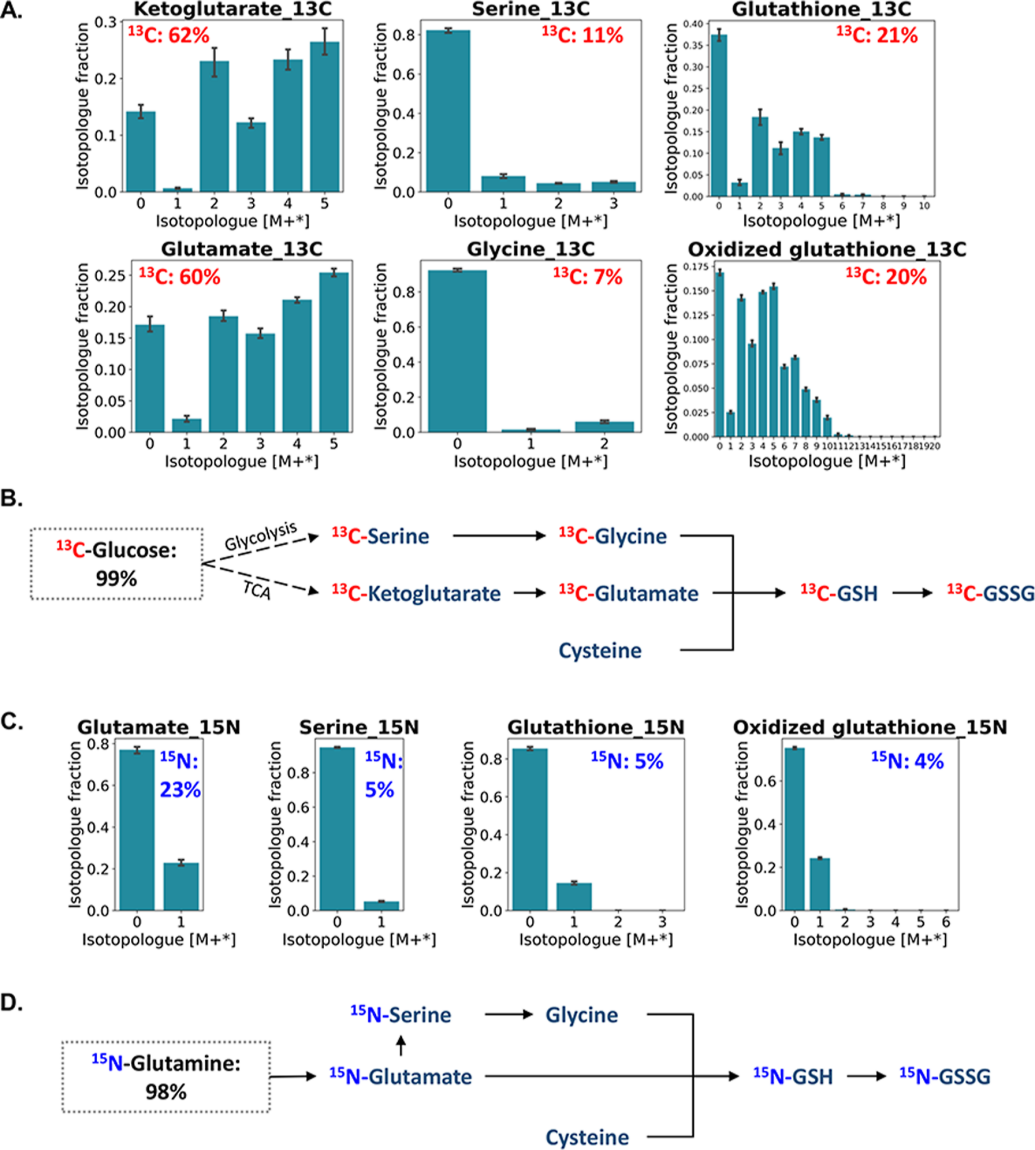
Tracing the de-novo glutathione synthesis pathway via ^13^C/^15^N atom enrichments of intermediate metabolites. The proportion of ^13^C/^15^N enrichment was denoted in red or blue. (A) ^13^C-labeled isotopologue distribution of intact metabolites. (B) ^13^C atom flow in the de-novo glutathione synthesis pathway using D-^13^C_6_-glucose as a carbon tracer. (C) ^15^N-labeled isotopologue distribution of intact metabolites. (D) ^15^N atom flow in the de-novo glutathione synthesis pathway using L-^15^N_2_-glutamine as a nitrogen tracer.

The analyzed intact isotopologues of key intermediate metabolites from healthy neurons were first used to decipher the key pathway connection associated with de novo glutathione synthesis. In Figure 5A, for healthy mid-brain neurons, intermediates of ketoglutarate and glutamate, and serine and glycine were detected at 62 and 60%, and 11 and 7% levels of ^13^C enrichment, respectively. GSH and GSSG showed 21 and 20% of ^13^C enrichment originating from D-^13^C_6_-glucose, respectively. The incorporation of ^13^C atoms from D-^13^C_6_-glucose into ketoglutarate and glutamate could be derived from the TCA cycle, and the ^13^C incorporation into serine and glycine could be derived from the de novo serine synthetic branch of glycolysis. The deciphered pathway reconstruction based on the ^13^C-enrichment of intermediates is shown in Figure 5B. Similarly, Figure 5C,D describes the pathway via ^15^N atom flow into de novo GSH synthesis. By tracing the ^15^N atoms from L-^15^N_2_-glutamine, 23 and 5% of ^15^N enrichment were found in glutamate and serine, while no ^15^N enrichment was detected in glycine. GSH and GSSG ultimately showed 5 and 4% of ^15^N enrichment originated from L-^15^N_2_-glutamine, respectively. To be noted, neither ^13^C nor ^15^N-labeling was found in cysteine, which suggested its independent supply from glucose or glutamine and instead a possible dependence on the essential uptake from the extracellular environment.

**Figure 6:**
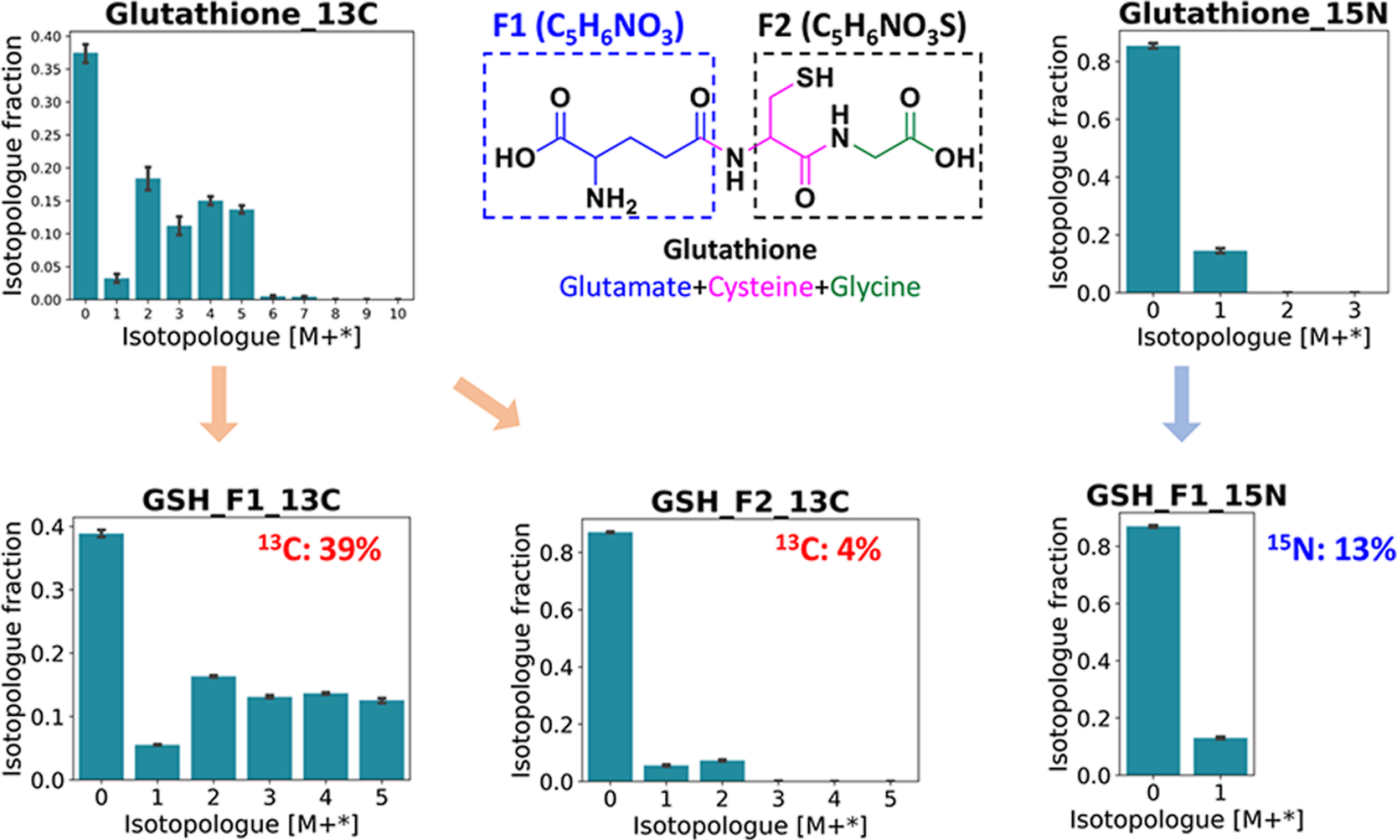
Structural elucidation of ^13^C/^15^N-labeled glutathione via fragment isotopologue analysis. Ionized glutathione could produce a mass spectrum of fragments by collision-induced dissociation, from which fragment 1 (F1) indicated a glutamate moiety and fragment 2 (F2) indicated a glycine–cysteine moiety.

Based on the intact isotopologues of ^13^C-GSH in M+1–7, we further investigated its fragment isotopologues (Figure 6); F1, indicating a glutamate moiety, was detected with a labeled distribution from m + 0 to m + 5 with 39% ^13^C enrichment, which is similar to the observed precursor glutamate MID pattern. In addition, F2, indicating a glycine–cysteine moiety, was detected with a labeled distribution from m + 0 to m + 2 and a ^13^C enrichment of just 4%. This was consistent with the corresponding patterns of the precursors glycine and cysteine. ^15^N-GSH was shown in M + 1 via the intact isotopologue analysis. The ^15^N enrichment is further observed only in the glutamate moiety (^15^N-GSH F1) with its MID shown from m + 0 to m + 1. This moiety labeling pattern also matched the precursor glutamate MID. Besides the fact that the precursor serine was detected with certain ^15^N enrichment, the level of ^15^N-glycine and its incorporation as ^15^N-GSH F2 could be too low to be detected. The fragment isotopologue distribution further validated the utilization of amino acid moieties derived from D-^13^C_6_-glucose/L-^15^N_2_-glutamine in the reconstructed pathway from Figure 5. Therefore, with the help of intact and fragment isotopologue analysis, we confirmed and highlighted that the de novo synthesis of GSH in mid-brain neurons requires both glucose and glutamine for providing de novo-synthesized glutamate, serine, or glycine.

**Figure 7:**
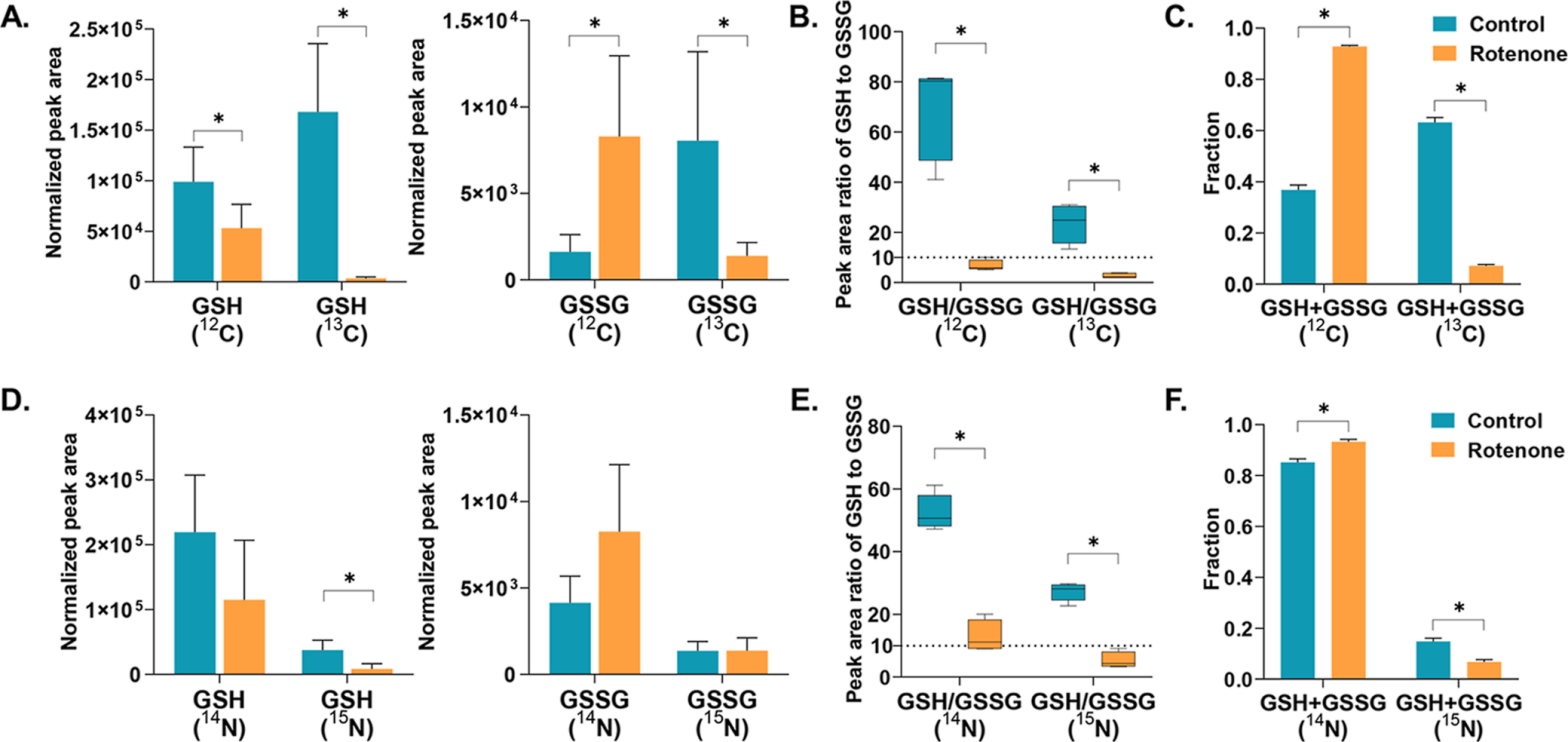
Metabolite abundance changes of GSH and oxidized glutathione (GSSG) under healthy and rotenone-treated conditions. (A) Bar plot representing the normalized peak area of ^12^C/^13^C GSH and GSSG. (B) Box plot representing the peak area ratio of ^12^C-GSH to ^12^C-GSSG and ^13^C-GSH to ^13^C-GSSG. (C) Bar plot representing the unlabeled (^12^C) and labeled (^13^C) fractions of combined GSH + GSSG. (D) Bar plot representing the normalized peak area of ^14^N/^15^N GSH and GSSG. (E) Box plot representing the peak area ratio of ^14^N-GSH to ^14^N-GSSG and ^15^N-GSH to ^15^N-GSSG. (F) Bar plot representing the unlabeled (^14^N) and labeled (^15^N) fractions of combined GSH + GSSG. Peak area was normalized using the corresponding sample protein content. An asterisk indicates a significant difference, with a *p*-value below 0.05.

Rotenone inhibits mitochondrial complex I, impairing oxidative phosphorylation and resulting in a dramatic reduction of ATP production. It also produces excess generation of reactive oxygen species and leads to decreased GSH levels.^44^ For mid-brain neurons with rotenone treatment, we detected decreased ^12^C-GSH and increased ^12^C-GSSG compared to controls (Figure 7A). Apart from the ^12^C-GSH pool, ^13^C-GSH and ^13^C-GSSG are synthesized de novo and both showed down-regulation. However, either the peak area ratio of ^12^C-GSH/^12^C-GSSG or the ratio of ^13^C-GSH/^13^C-GSSG was significantly decreased below 10 due to rotenone-induced oxidative stress (Figure 7B), which is consistent with a previous report.^45^ Reduced peak area ratios of ^14^N-GSH/^14^N-GSSG and ratios of ^15^N-GSH/^15^N-GSSG were also found in ^15^N-labeled neurons (Figure 7D,E). A low GSH/GSSG ratio, as a result of antioxidant defense, may act as a critical factor in the neuroinflammatory and neurodegenerative processes in Parkinson’s disease.^46^ Interestingly, rotenone treatment also resulted in a significantly decreased labeled (^13^C/^15^N) fraction of the combined GSH + GSSG (Figure 7C,F), further implying defective GSH biosynthesis in rotenone-treated neurons.

**Figure 8:**
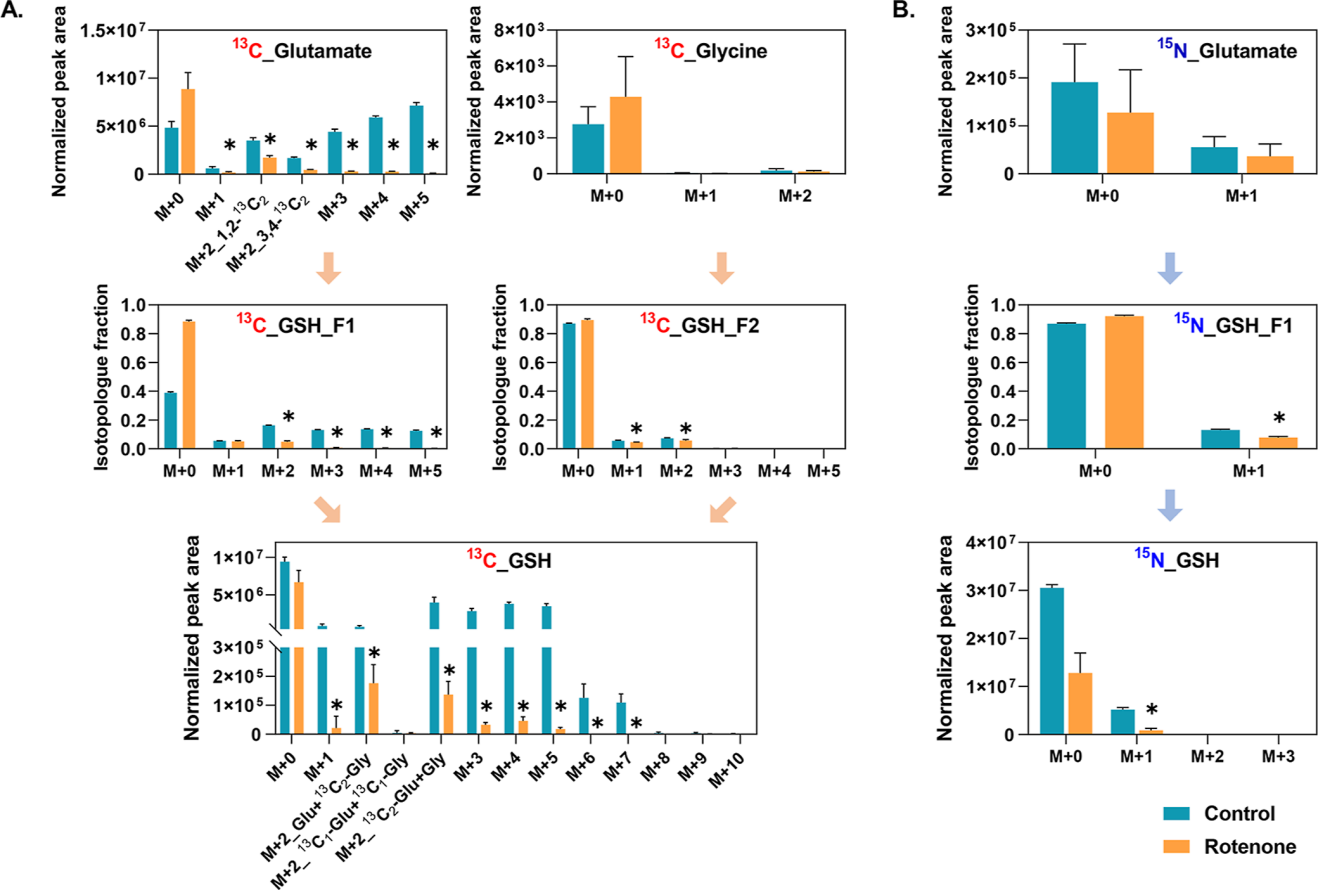
Mass isotopologue analysis of intact and fragmented metabolites for healthy and rotenone-perturbed mid-brain neurons. (A) ^13^C-labeled isotopologue analysis of glutamate, GSH, and its moieties F1 and F2. The M + 2 isotopologue of ^13^C-glutamate was identified in two isotopomers. The M + 2 isotopologue of ^13^C-GSH was identified in three isotopomers. (B) ^15^N-labeled isotopologue analysis of glutamate, GSH, and its moiety F1. An asterisk indicates a significant difference, with a *p*-value below 0.05.

To figure out the cause of low glutathione synthesis through de novo regulation, we next analyzed the ^13^C/^15^N-labeling patterns of both the intact molecule and its moieties for GSH and the associated intermediates (Figure 8). Rotenone induced significant depletion in both 1,2–^13^C_2_-glutamate and 3,4–^13^C_2_-glutamate, which pointed to the inhibition of PDH and PC-mediated TCA cycle activity. Additionally, the ^13^C-glutamate moiety of GSH (^13^C-GSH F1) showed a decreased ^13^C fraction. This confirmed that rotenone reduced glutamate production by inhibiting the entry flux into the upstream TCA cycle, rather than increasing its consumption for downstream synthesis. No significant depletion was observed in ^13^C-glycine, while the ^13^C-glycine moiety of GSH (^13^C-GSH F2) showed a significantly decreased ^13^C fraction after rotenone treatment. Based on the distribution ratio between m + 0, m + 1, and m + 2 of F1 isotopologues (Table S8), a tandem analysis of ^13^C-GSH including three positional isotopomers for the M + 2 isotopologue was obtained, as shown in Figure 8A. In line with the reduced ^13^C enrichment found in GSH moieties F1 and F2, the abundance of two major isotopomers, ^13^C_2_-GSH: M + 2_Glu + ^13^C_2_-Gly and M + 2_^13^C_2_-Glu + Gly, decreased significantly in rotenone-treated conditions. Similar to the alterations of ^13^C-glycine and ^13^C-GSH F2, no change was found in ^15^N-glutamate in the rotenone-treated group, while the ^15^N-glutamate moiety of GSH (^15^N-GSH F1) showed a significantly decreased ^15^N fraction, and ^15^N-GSH showed corresponding decreases in abundance (Figure 8B).

The results of mass isotopologue analysis showed that, in addition to suppressing glucose metabolism, which directly limits the source of glutamate supplied for de novo GSH synthesis, rotenone may also cause an inhibitory effect on the synthetic reaction of GSH production from glutamate, cysteine, and glycine. The sequential reactions are catalyzed by ATP-dependent enzymes γ-glutamylcysteine synthetase (γ-GCS) and GSH synthetase (GS). Perceived flux reduction of reactions catalyzed by PDH, PC, γ-GCS, and GS may be a subsequent effect of mitochondrial complex I inhibition, which will need future validation to better understand metabolic dysfunction during rotenone-induced neurodegeneration. Overall, our results suggest that in this neuronal model of rotenone-induced neurodegeneration, deficient de novo GSH synthesis and increased oxidation into GSSG together resulted in a decreased GSH level under oxidative stress.

## Conclusions

In this study, we developed a HILIC-Zeno MRM^HR^ method that can be used in tracer-based metabolomics studies for structurally resolved MID analysis. This method allows simultaneous acquisition at MS^1^ and MS^2^ levels in one single run. Labeled isotopologue distributions for intact metabolites can be obtained from the MS^1^ level. Meanwhile, labeled isotopologue distributions for both the intact metabolite and its fragmented moieties can be obtained from the MS^2^ level with higher sensitivity due to Zeno trap pulsing. The relationship between the labeled precursor and fragment ions was preserved to accurately identify the same labeled isotopologue with differential labeling positions. For future work, intensity-dependent selection of precursor ions can be combined with the Zeno trap to trigger MS^2^ for only present isotopologues, thus achieving even higher sensitivity. Furthermore, including additional target metabolites would provide more insights into pathway regulation, such as for γ-glutamylcysteine.

The method was successfully applied to analyze ^13^C/^15^N-labeled polar extracts of human-derived mid-brain neurons under healthy and oxidatively stressed states using D-^13^C_6_-glucose/L-^15^N_2_-glutamine as tracers. By tracing the labeled ^13^C/^15^N atoms in the moieties of metabolite isotopologues, we were able to reconstruct the cell-type and condition-specific pathways of glutathione metabolism in healthy and perturbed mid-brain neurons. The quantitative isotopologue analysis greatly contributed to the new elucidation of glutathione metabolism regulation in response to rotenone perturbation. It is worth mentioning that quantitative isotopologue analysis highlights altered metabolic fluxes, providing guidance for the subsequent targeted analysis of changes in enzymatic activities, which expands our understanding of disease mechanisms at the enzyme level. Although we only present the application of our approach to glutathione metabolism, it can also be applied to study other pathways including central carbon metabolism and de novo nucleotide metabolism. Thereby, more accurate biological interpretations could be achieved within a cell-specific metabolic network.

## Supporting Information Available

SWATH acquisition method optimization and evaluation; extracted ion chromatography of target metabolites; peak scan point illustration; linearity test of glutamate and glutathione based on the quantification for TOF-MS and MS/MS levels; sensitivity comparison among SWATH, MRM^HR^, and Zeno MRM^HR^ acquisition for ^15^N-labeled isotopologue analysis; accuracy comparison between SWATH, MRM^HR^, and Zeno MRM^HR^ acquisition for ^15^N-labeled isotopologue distribution analysis; and product ion fragment annotation of glutamate and glutathione (PDF)
Target metabolite list; Q1 mass isolation window setting design for SWATH acquisition method optimization; inclusion precursors covered in the ^13^C-labeling MRM^HR^ acquisition method; inclusion precursors covered in the ^15^N-labeling MRM^HR^ acquisition method; analysis of calibration curves by linear regression for targeted polar metabolites; reproducibility evaluation of Zeno MRM^HR^ acquisition for ^13^C MID analysis; and determination of isotopomer distribution for ^13^C_2_-glutamate and ^13^C_2_-GSH (XLSX)
