# Supplementary material for: Reconstruction of Glutathione Metabolism in the Neuronal Model of Rotenone-Induced Neurodegeneration Using Mass Isotopologue Analysis with Hydrophilic Interaction Liquid Chromatography-Zeno High-Resolution Multiple Reaction Monitoring
Source: Anal Chem. 2023 Feb 3;95(6):3255–66. doi: 10.1021/acs.analchem.2c04231 (PMC9933045; doi:10.1021/acs.analchem.2c04231)
Supplement: Supplementary file 1 — ac2c04231_si_001.pdf [file ac2c04231_si_001.pdf]

Supporting information

Reconstruction of Glutathione Metabolism in the Neuronal Model of Rotenone-Induced Neurodegeneration Using Mass Isotopologue Analysis with Hydrophilic Interaction Liquid Chromatography-Zeno High-Resolution Multiple Reaction Monitoring

Luojiao Huang[1], Nicolas Drouin[1], Jason Causon[2], Agnieszka Wegrzyn[1], Jose Castro-Perez[2], Ronan Fleming[1, 3], Amy Harms[1], Thomas Hankemeier[1, *]

1. Metabolomics and Analytics Centre, Leiden Academic Centre for Drug Research, Leiden University, Leiden, 2333 CC, Netherlands

2. SCIEX, Concord, Ontario L4K 4V8 Canada

3. School of Medicine, National University of Ireland, University Rd, Galway, H91 TK33, Ireland.

***** Corresponding author: Thomas Hankemeier, Professor of Metabolomics and Analytics Centre, Leiden Academic Centre for Drug Research, Leiden University, Leiden, 2333 CC, Netherlands Email: hankemeier@lacdr.leidenuniv.nl, Tel: +31-715274226

Table of Contents

1. Optimal mass isolation window setting for SWATH acquisition method 3

Figure S1. Peak integration influence on target metabolite isotopologues spanning across two neighboring SWATH mass isolation windows compared to complete isotopologues detection within a single window... 4

Figure S2. Extracted ion chromatography of target metabolites. 5

Figure S3. A. Illustration of peak scan points for metabolites glutamate and glutathione eluting between 4 and 6 mins, using ^{13}C labeling Zeno MRM^{HR} acquisition method; B. Linearity test of glutamate and glutathione based on the quantification for TOF-MS and MS/MS level. 5

Figure S4. Sensitivity comparison at MS^1 TOF level and MS^2 fragmentation level among SWATH, MRM^{HR} and Zeno MRM^{HR} acquisition for ^{15}N labeled isotopologue analysis.. 6

Figure S5. Accuracy comparison at MS^1 TOF level and MS^2 fragmentation level between SWATH, MRM^{HR} and Zeno MRM^{HR} acquisition for ^{15}N labeled isotopologue distribution analysis..................... 6

Figure S6. Product ion fragment annotation of glutamate.. 7

Figure S7. Product ion fragment annotation of glutathione... 7

References... 7

1. Optimal mass isolation window setting for SWATH acquisition method

When optimizing a SWATH acquisition method, considerations need to be made defining scan time, window size and total number of windows. Additional considerations need to be made in tracer-based studies especially when analyzing diverse metabolites. In this case, for accurate quantitation, all isotopologues of a certain metabolite need to be in the same Q1 window and care should be taken to prevent the underestimated integration of the mass isotopologue located on the edge of a SWATH window.[1] This experiment was designed to test the effect of complete or partial isotopologue coverage in one Q1 window, as well as the influence of overlapping windows giving partial isotopologue coverage. In total four SWATH methods were created with varied Q1 isolation windows and window overlap, as shown in Table S2. Each SWATH method starts with a survey TOF MS scan in 100 ms from 50 to 700 Da, followed by sixteen Q1 isolation windows covering a mass range of *m/z* 60-690. The relevant Q1 isolation windows in each SWATH method targeting ^{13}C labeled glutamate were listed in Figure S1, also recorded in cycleID of 2 and 3 from Table S2. SWATH_Win1 and Win4 methods allow glutamate isotopologues of M0-M5 to be measured and quantified in the same window, but SWATH_Win2 and Win3 methods measured and quantified isotopologues across the two neighboring windows.

As a result, SWATH_Win1 and Win4 methods shared similar isotopologue quantification on both isotopologue peak area and isotopologue fraction which was also comparable to the results of general MRM^{HR} acquisition. However, the SWATH_Win2 method had lower signal for the M0-M2 isotopologues and this led to a change in their mass isotopologue fractions. The increased window overlapping width of 3.0 Da in the SWATH_Win3 method showed no improvement in isotopologue quantification combining neighboring windows. The results indicated a big drawback for the quantification of isotopologues of metabolites that span multiple windows, which would also cause subsequent inaccuracies in the quantification of fragment isotopologues. For targeted labeled metabolite analysis, it is strongly recommended to refine the Q1 window setting to include all possible isotopologues of precursor ions, even with the demand of making a custom SWATH window program or creating variable Q1 isolation windows.[1,2] In this study, the SWATH_Win1 method with fixed Q1 isolation window was finally selected for the formal comparison with general MRM^{HR}, and Zeno MRM^{HR} acquisition.

SWATH method	CycleID	Overlap window width	*m/z* begin	*m/z* end	Quantified mass isotopologue
SWATH_Win1	2	1.0 Da	99	140	\
	3		139	180	M0,M1,M2,M3,M4,M5
SWATH_Win2	2	1.0 Da	99	149	**M0,M1,M2**
	3		148	180	M3,M4,M5
SWATH_Win3	2	3.0 Da	97	149	**M0**
	3		146	180	M1,M2,M3,M4,M5
SWATH_Win4	2	5.0 Da	95	149	\
	3		144	180	M0,M1,M2,M3,M4,M5

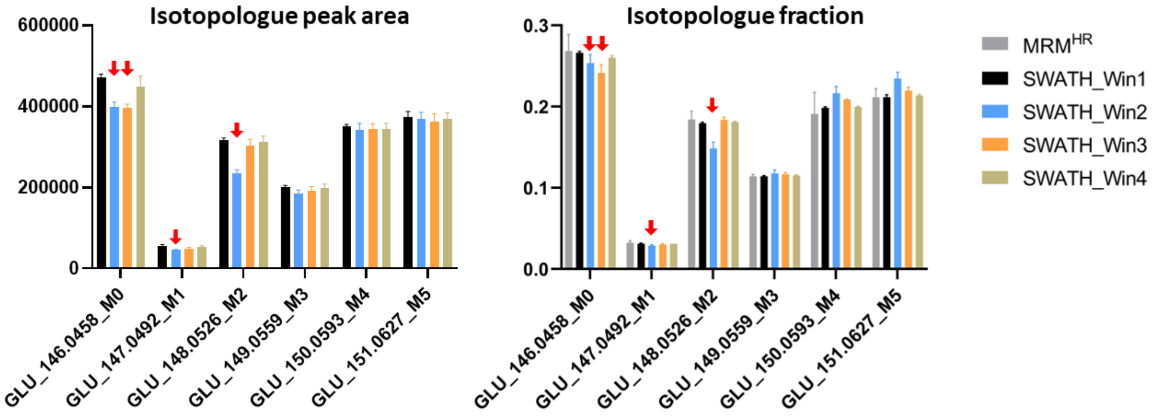

Figure S1. Peak integration influence on target metabolite isotopologues spanning across two neighboring SWATH mass isolation windows compared to complete isotopologues detection within a single window.

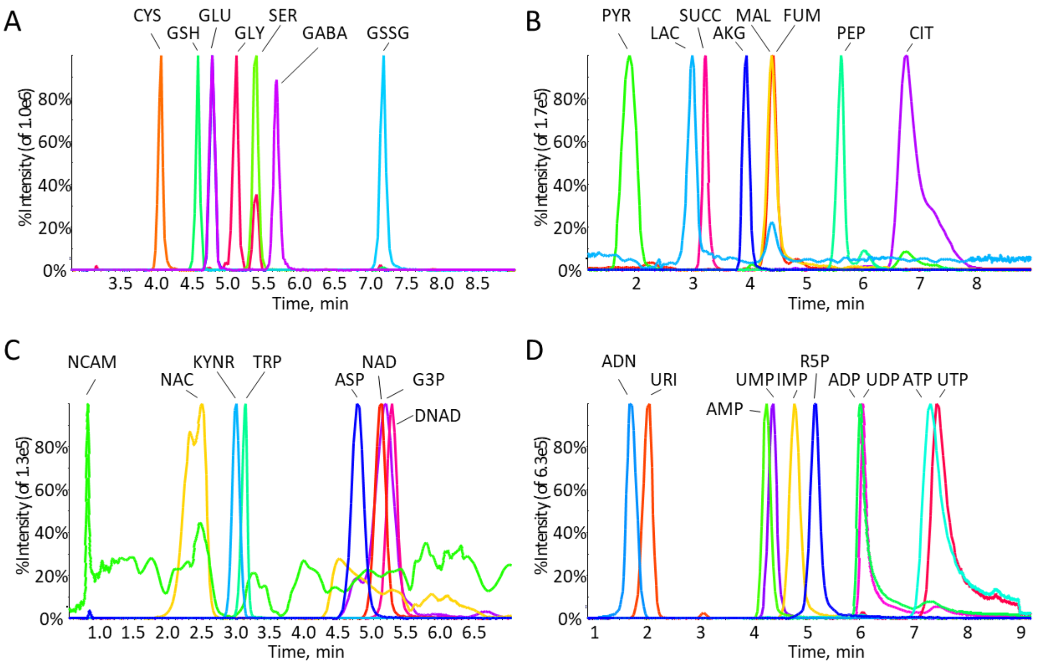

Figure S2. Extracted ion chromatography of target metabolites from de novo glutathione synthesis (A), primary carbon metabolism (B), de novo NAD synthesis (C), purine and pyrimidine metabolism (D). The metabolite abbreviations were introduced in Table S1.

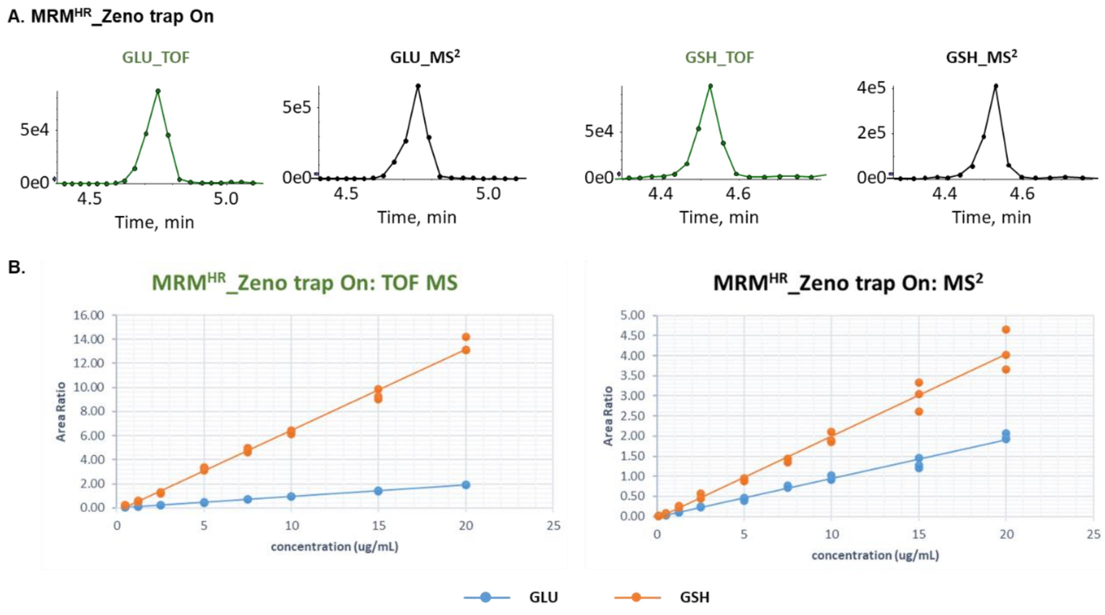

Figure S3. A. Illustration of peak scan points for metabolites glutamate (GLU) and glutathione (GSH) eluting between 4 and 6 mins, using ^{13}C labeling Zeno MRM^{HR} acquisition method; B. Linearity test of glutamate (GLU) and glutathione (GSH) based on the quantification for TOF-MS and MS/MS level.

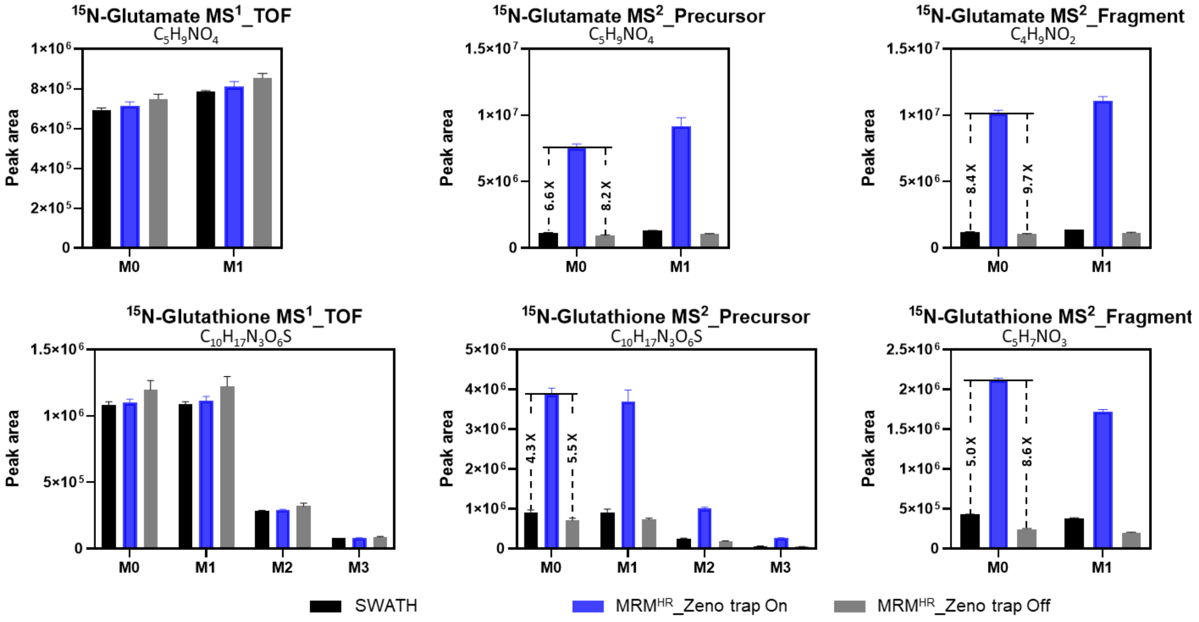

Figure S4. Sensitivity comparison at MS[1] TOF level and MS[2] fragmentation level among SWATH, MRM^{HR} and Zeno MRM^{HR} acquisition for ^{15}N labeled isotopologue analysis (n=3). At the MS[2] level, each precursor isotopologue was quantified using the peak area of residual precursor ion extracted from its MS/MS scan window. Each fragment isotopologue was quantified by summing the peak areas of the same fragment ion extracted from multiple MS/MS scan windows.

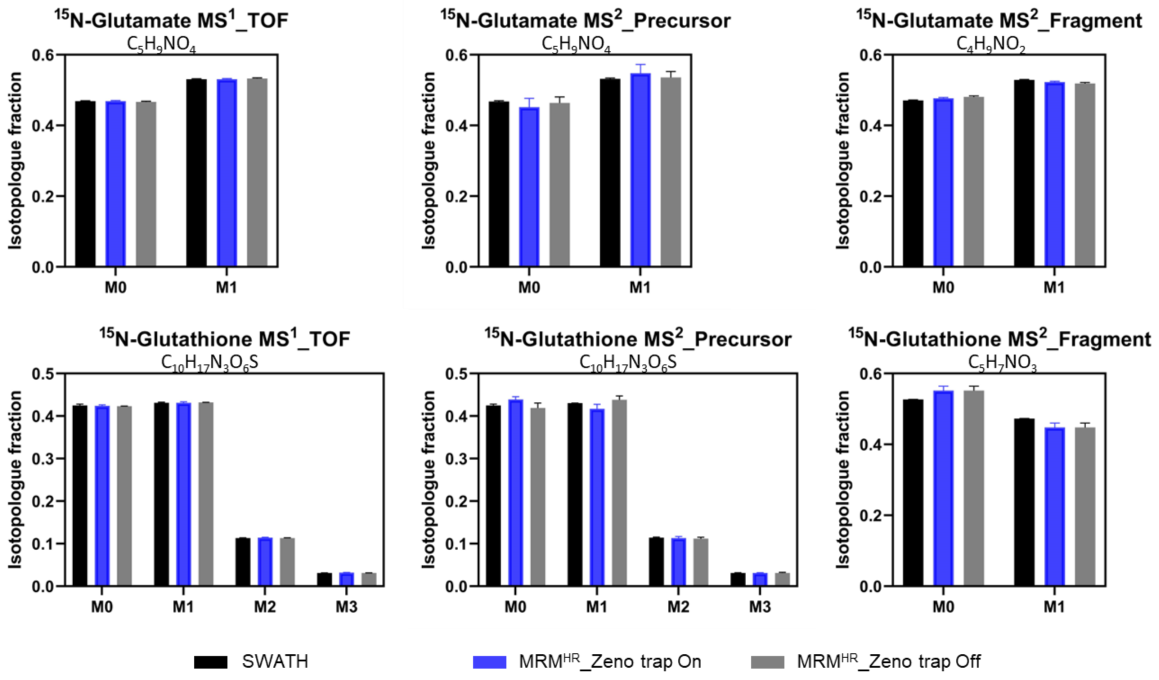

Figure S5. Accuracy comparison at MS[1] TOF level and MS[2] fragmentation level between SWATH, MRM^{HR} and Zeno MRM^{HR} acquisition for ^{15}N labeled isotopologue distribution analysis (n=3).

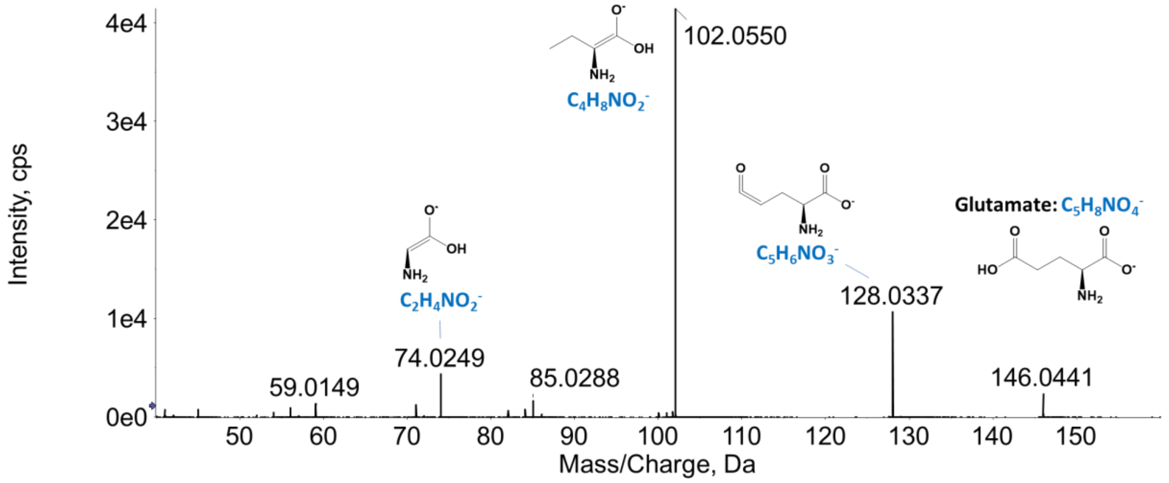

Figure S6. Product ion fragment annotation of glutamate.

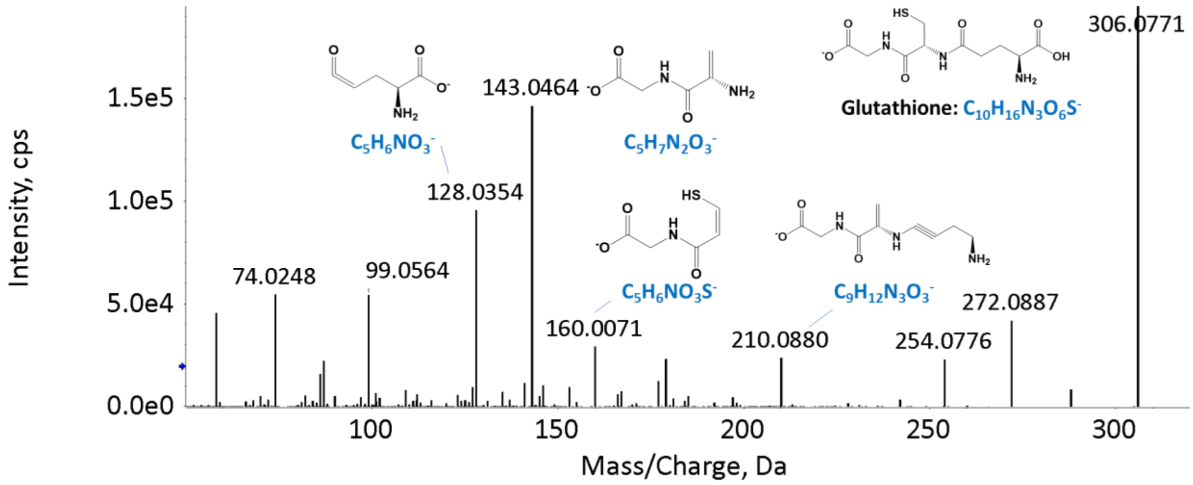

Figure S7. Product ion fragment annotation of glutathione.

References

(1) Jaiswal, D.; Prasannan, C. B.; Hendry, J. I.; Wangikar, P. P. SWATH Tandem Mass Spectrometry Workflow for Quantification of Mass Isotopologue Distribution of Intracellular Metabolites and Fragments Labeled with Isotopic ^{13}C Carbon. *Analytical Chemistry* **2018**, *90* (11), 6486–6493.

(2) Zhang, Y.; Bilbao, A.; Bruderer, T.; Luban, J.; Strambio-De-Castillia, C.; Lisacek, F.; Hopfgartner, G.; Varesio, E. The Use of Variable Q1 Isolation Windows Improves Selectivity in LC–SWATH–MS Acquisition. *J. Proteome Res.* **2015**, *14* (10), 4359–4371.
